# Infection in Health Personnel with High and Low Levels of Exposure in a Hospital Setting during the H1N1 2009 Influenza A Pandemic

**DOI:** 10.1371/journal.pone.0147271

**Published:** 2016-01-22

**Authors:** Carmen Sandoval, Aldo Barrera, Marcela Ferrés, Jaime Cerda, Javiera Retamal, Adolfo García-Sastre, Rafael A. Medina, Tamara Hirsch

**Affiliations:** 1 Departmento de Enfermedades Infecciosas e Inmulogia Pediátrica, Escuela de Medicina, Pontificia Universidad Católica de Chile, Santiago, Chile; 2 Laboratory of Infectious Diseases and Molecular Virology, Centro de Investigaciones Médicas, Escuela de Medicina, Pontificia Universidad Católica de Chile, Santiago, Chile; 3 Public Health Department, Pontificia Universidad Católica de Chile, Santiago, Chile; 4 Department of Microbiology and Global Health and Emerging Pathogens Institute, Icahn School of Medicine at Mount Sinai, New York, NY, United States of America; 5 Department of Medicine, Icahn School of Medicine at Mount Sinai, New York, NY, United States of America; 6 Instituto Milenio en Inmunología e Inmunoterapia, Pontificia Universidad Católica de Chile, Santiago, Chile; 7 Hospital Clínico, Emergency Room, Escuela de Medicina, Pontificia Universidad Católica de Chile, Santiago, Chile; 8 Departamento de Pediatría, División de Pediatría, Escuela de Medicina, Pontificia Universidad Católica de Chile, Santiago, Chile; University of Hong Kong, HONG KONG

## Abstract

A novel H1N1 influenza A virus caused the first pandemic of the 21^st^ century in 2009. Hospitals had an increased demand of health consultations, that made it difficult to estimate the incidence of infection in hospital personnel due to asymptomatic presentations and the under notification of cases. To estimate and compare the rate of exposure of high versus low risk health personnel to 2009 pandemic H1N1 (H1N1pdm2009) influenza A virus in a University Hospital in Chile, we performed a comparative and prospective study. Serum samples were obtained from 117 individuals that worked in the emergency room (ER) and the operating room (OR) during the peak of the pandemic. Antibody titers were determined by the hemagglutination inhibition (HI) assay. Of the samples analyzed, 65% were workers at the ER and 35% at the OR. Of the total number of the subjects tested, 29.1% were seropositive. One out of 3 (36.8%) workers at the ER had positive HI titers, meanwhile only 1 out of 7 (14.6%) workers from the OR was seropositive to the virus. The possibility of being infected in the ER as compared to the OR was 3.4 times greater (OR 3.4; CI 95%, 1.27–9.1), and the individuals of the ER had almost twice as much antibody titers against H1N1pdm2009 than the personnel in the OR, suggesting the potential of more than one exposure to the virus. Of the 34 seropositive subjects, 12 (35.3%) did not develop influenza like illness, including 2 non-clinical personnel involved in direct contact with patients at the ER. Considering the estimated population attack rate in Chile of 13%, both groups presented a higher exposure and seropositive rate than the general population, with ER personnel showing greater risk of infection and a significantly higher level of antibodies. This data provide a strong rationale to design improved control measures aimed at all the hospital personnel, including those coming into contact with the patients prior to triage, to prevent the propagation and transmission of respiratory viruses, particularly during a pandemic outbreak.

## Introduction

During April 2009 the authorities of the World Health Organization (WHO) emitted the alert of the emergence of a novel H1N1 influenza A virus affecting humans in Mexico and the Southern United States [1]. Soon after, the WHO declared the first influenza pandemic of the 21^st^ century. The emergency departments of hospitals in many countries had to face an abrupt increase in the demand of healthcare visits; a scenario that highly increased the risk of exposure of the health personnel to this pandemic virus [1]. Estimations of the incidence of infection in hospital personnel has been difficult, particularly due to under notification of cases and poor estimations of hospitalization rates, in addition to low seroconversion rates and asymptomatic cases [2].

In October 2009 the WHO reported that the asymptomatic infection rate of this virus had reached 9%, and that if asymptomatic infection reached health personnel it would transform this population in a high-risk transmission group [3, 4]. Other studies have investigated the seropositivity of health care workers (HCW) to the pandemic H1N1 2009 (H1N1pdm2009) influenza A virus, demonstrating that this population, with a higher exposure to infected patients, presented increased seropositive rates, ranging from 5.25–25.1% in different clinical settings in Asia, Europe, Australia and the United states, as compared to those the general population [5–15]. In addition, a comparison amongst health staff at different clinical departments during the first wave (August-September) of the 2009 H1N1 pandemic in Spain, demonstrated that personnel working at the Emergency Room (ER) had the highest seropositivity (36.6%) of all health workers tested [11]. In contrast, a different study conducted during the first wave (April-June) in the United States, revealed that personnel working in acute care units or designated influenza areas, did not show an increased risk of influenza infection [16]. A direct comparison of risk exposure and seropositivity rates of HCW has not been fully addressed. Thus, additional studies are needed to further understand the specific occupational risk for influenza infection in healthcare personnel in diverse clinical settings, particularly during a pandemic setting

While the H1N1pdm2009 virus emerged during the spring in the Northern Hemisphere, the first wave of the outbreak in Chile occurred on weeks 20 to 33 (from the second week of May to the second week of August) during the winter season of the Southern hemisphere; at a time when other seasonal respiratory viruses also circulated in the population. Thus, the dynamics and rate of infection likely differed from those of the Northern hemisphere. This highlights the value of estimating the rate of influenza virus infection in health personnel in this region, in order to establish improved global prevention strategies aimed at reducing the infection and transmission rates from health personnel to patients and other members of the health community in a pandemic setting [4].

In this study we determined the seropositivity rates and antibody titers against the novel H1N1pdm2009 influenza A strain of healthcare professionals in the context of a University Hospital in Santiago during the pandemic outbreak. We estimated and compared the infection rate of high versus low risk health personnel during the outbreak by comparing the seroprevalence against this virus versus the clinical definition of disease. In addition, we evaluated the adherence of health workers to the preventive measures for infection control, as recommended the United States Centers for Disease Control and Prevention (CDC), and its effectiveness in preventing occupational exposures to influenza.

## Materials and Methods

### Study design and clinical samples

We determined seroprevalence at the Pontificia Universidad Catolica Clinical Hospital during the first wave of the H1N1pdm2009 Influenza A. The samples were obtained at least 3 weeks after the end of the pandemic period (beginning in the last week of November of 2009), and before the vaccination campaign against the H1N1pdm2009 virus that begun during March of 2010. This study was reviewed and approved by the Scientific Ethics Committee of the School of Medicine at Pontificia Universidad Católica de Chile under protocol number 09–203. These samples were archived under approved protocols after informed written consent was obtained. All the samples used were deidentified of any personal information. The estimated target sample size of our study was determined to be 206 individuals (103 per group). For this calculation we considered the original reports estimating a 13% exposure of the general population in Chile [17]. Thus, during the main wave of the pandemic outbreak we estimated that the potential for general health personnel in our hospital of being exposed and becoming positive to the virus was approximately 50%, considering a confidence interval of 95% and a power of 80%. Recruitment was done on a voluntary basis where all health personnel from the Emergency Room personnel (ER) and Operating Room (OR) were invited to participate in the study, without any exclusions or apparent bias observed during the recruitment process. During the study the total number of HCW working in the ER were 140 people and in the OR there were 120 people. Informed consent and a clinical-epidemiological survey for both groups were obtained during the same period, along with a blood sample for determining seropositivity and antibody titers against the H1N1pdm2009 Influenza A virus. Overall we recruited and obtained samples from 117 individuals that worked during the peak of the H1N1pdm2009 Influenza A virus outbreak our University Clinical Hospital, which occurred between epidemiologic weeks 20 to 33 (May to August of 2009). 76 of the participants were ER (considered to be a group at high risk of exposure to the virus) and 41 worked at the OR (considered to be at low risk of exposure group), corresponding to 54.3% and 34.2% of the total ER and OR personnel, respectively.

The experiments conducted to assess the reactivity of these human sera against H1N1pdm2009 were performed in duplicate at the Microbiology Department at Icahn School of Medicine at Mount Sinai, New York. To assess the reactivity of the sera to the seasonal strains A/Brisbane/59/2007(H1N1) and the A/Brisbane/10/2007(H3N2), the assays were repeated at the Laboratory of Molecular Virology at Pontificia Universidad Católica de Chile, Santiago, yielding virtually identical results. HI assays were performed blinded following the exact same protocol [18] [19]. All sera were tested at the same time when analyzed against each virus strain, utilizing the same reagents, under the same experimental and laboratory conditions.

### Hemagglutination inhibition (HI) assay

The sera were inactivated by the trypsin-heat-periodate treatment as previously described [18]. Briefly, the sera was mixed with half a volume of trypsin 8 mg/ml (Sigma-Aldrich, St. Louis, MO) diluted in 0.1 M phosphate buffer, pH 8.2 followed by an incubation for 30 min at 56°C. The samples were then mixed with 3 volumes of 0.11 M metapotassium periodate and incubated at room temperature (RT) for 15 min. Three volumes of 1% glycerol saline were then added and mixed with the samples and incubated for and additional 15 min at RT. Finally, the samples were brought to a final 1:10 dilution by mixing with 2.5 volumes of 85% saline. We performed HI assays of the treated sera by following standard protocols [19]. Two-fold serial dilutions of sera were mixed in 96-well plates with 8 HA units of virus per well and were incubated at 4°C for 30 min. Turkey red blood cells were then added to a final concentration of 0.25%, and the plate was incubated for and additional 30 min at 4°C. HI titers of sera were determined as the highest dilution that displayed hemagglutinating activity. Individuals were considered to be seropositive if they had a serum titer of ≥20 HI units.

### Statistical analysis

Categorical variables were expressed as number and percentage. We collected data on the following categorical variables: sex, workplace, seropositivity, influenza vaccination (2009 pre-pandemic trivalent vaccine), hand hygiene, use of clinical mask and occupation. Continuous variables (i.e. age) were described in terms of median and range, and then compared by the Mann-Whitney test. We constructed a logistic regression model, which estimated unadjusted and adjusted odds ratios (OR) of seropositivity and 95% confidence intervals. The goodness of fit of the model was evaluated by the Hosmer and Lemeshow Test. For the analysis of antibody titers, we used an Unpaired t-test with Welch's correction and the F Test was used to evaluate variances (*p* 0.0001). All the statistical analyses were made with the SPSS 15.0 software. Statistically significance was determined when *p* < 0.05.

## Results

### Personnel of the ER had a higher incidence infection and magnitude of antibody titers during the H1N1pdm2009 outbreak

The samples included 117 individuals, 76 of them worked at the ER (65.0%) and 41 at the OR (35.0%). The median age in each group was 37.5 years (22−64) and 36 years (range 21–60), respectively (p = 0.732).

Of the total number of the subjects tested, 34/117 (29.1%) were seropositive by HI assay. Of these, 36.8% (28/76) of the workers at the ER had positive HI titers, meanwhile only 14.6% (6/41) of the workers at the OR were seropositive (Table 1). Expressed in relative terms, the chance of seropositivity in the ER as compared to the OR was 3.4 times greater (OR 3.40; CI 95%, 1.27–9.10) (Table 2). Personnel from the ER and OR, reported a similar levels of compliance to hand hygiene (84 and 86%, respectively), however, there was a statistical significant difference when it came to the use of clinical mask, which only had a 34.7% adherence in the ER and a 87.8% in the OR (p < 0.001). When adjusting the comparison between these groups by the variables “2009 seasonal influenza vaccination”, “hand hygiene” and “clinical mask use”, the odds ratio of positive HI titers for pH1N1 2009 increased to 4.1 (adjusted OR 4.14; CI 95%, 1.24–13.86) between the staff members of ER versus OR. The goodness of fit of the model was optimal (Hosmer-Lemeshow p-value = 0.716). It is important to mention that having received the 2009 seasonal trivalent influenza vaccine, performing proper hand hygiene and wearing a clinical mask was associated to a lower risk of seropositivity (Table 2).

**Table 1:** Seropositivity Distribution of Operating Room and Emergency Room personnel according to their occupation.

Occupation	Operating Room*		Emergency Room*		Total (%)**
	Number (%)	Seropositive (%)	Number (%)	Seropositive (%)	
**Nurse**	10 (24.4)	0	22 (29.7)	8 (10.8%)	32 (27.4)
**Paramedic**	21 (51.2)	3 (7.3%)	14 (18.9)	4 (5.4%)	35 (29.9)
**Theater Nurse**	5 (12.2)	1 (2.4%)	−	0	5 (4.3)
**Janitor**	2 (4.9)	1 (2.4%)	7 (9.5)	4 (5.4%)	9 (7.7)
**Clerk**	2 (4.9)	1 (2.4%)	8 (10.8)	3 (4.0%)	10 (8.5)
**Physician**	1 (2.4)	0	20 (27.0)	7 (9.5%)	21 (17.9)
**Security Guard**	−	0	3 (4.1)	2 (2.7%)	3 (2.6)
**N/A**	−	-	2	0	2 (1.7)
**Total**	41 (100)	6 (14.6)	76 (100)	28 (36.8)	117

* Percentages are calculated from the total number of personnel in each group.

** Percentage of total personnel

N/A: Data not available.

**Table 2:** Proportion of influenza A pH1N1 2009 seropositive individuals, listed by variable.

Variable	Positive Serology, Number (%)	OR unadjusted (IC95%)	OR adjusted (IC95%)**
**Place of Work**			
** ER**	28/76 (36.8)	3.40 (1.27−9.10)	4.14 (1.24−13.86)
** OR**	6/41 (14.6)	1.00	1.00
**2009 Seasonal Vaccine**			
** No**	10/26 (38.5)	1.69 (0.68−4.24)	3.02 (1.02−8.92)
** Yes**	24/89 (27.0)	1.00	1.00
**Hand Washing**			
** Poor or never**	8/18 (44.4)	2.19 (0.79−6.14)	2.52 (0.74−8.64)
** Always**	26/97 (26.8)	1.00	1.00
**Use of masks**			
** Poor or never**	21/53 (39.6)	2.37 (1.04−5.41)	1.21 (0.43−3.36)
** Always**	13/60 (21.7)	1.00	1.00

** Model included the four variables listed.

In relation to the presence of clinical symptoms, when considering all of the 34 seropositive subjects, 12 (35.3%) did not develop influenza like illness (ILI; 9 worked at ER and 3 at OR). Two out of these 12 subjects did not perform direct clinical care, but were involved in attention to general public (a clerk and a security guard). Among the 34 seropositive individuals, 4 subjects had high HI titers of ≥ 160, and all of them worked at the ER. From this group, only one subject was asymptomatic (the security guard) and the other three presented ILI. Fifty percent (11/22) of the personnel that did not perform direct clinical duties had positive serology.

Considering the entire sampling during the study period, none of the subjects were hospitalized. Out of the entire workforces (ER and OR), a total of 14 health related absentees occurred due to ILI, of these 7 were HI positive staff that worked at the ER. Finally, 27.3% (32/117) of subjects reported contact with sick household members, of which 18 had ILI but only 4 had positive HI titers against the H1N1pdm2009 strain.

**Fig 1:**
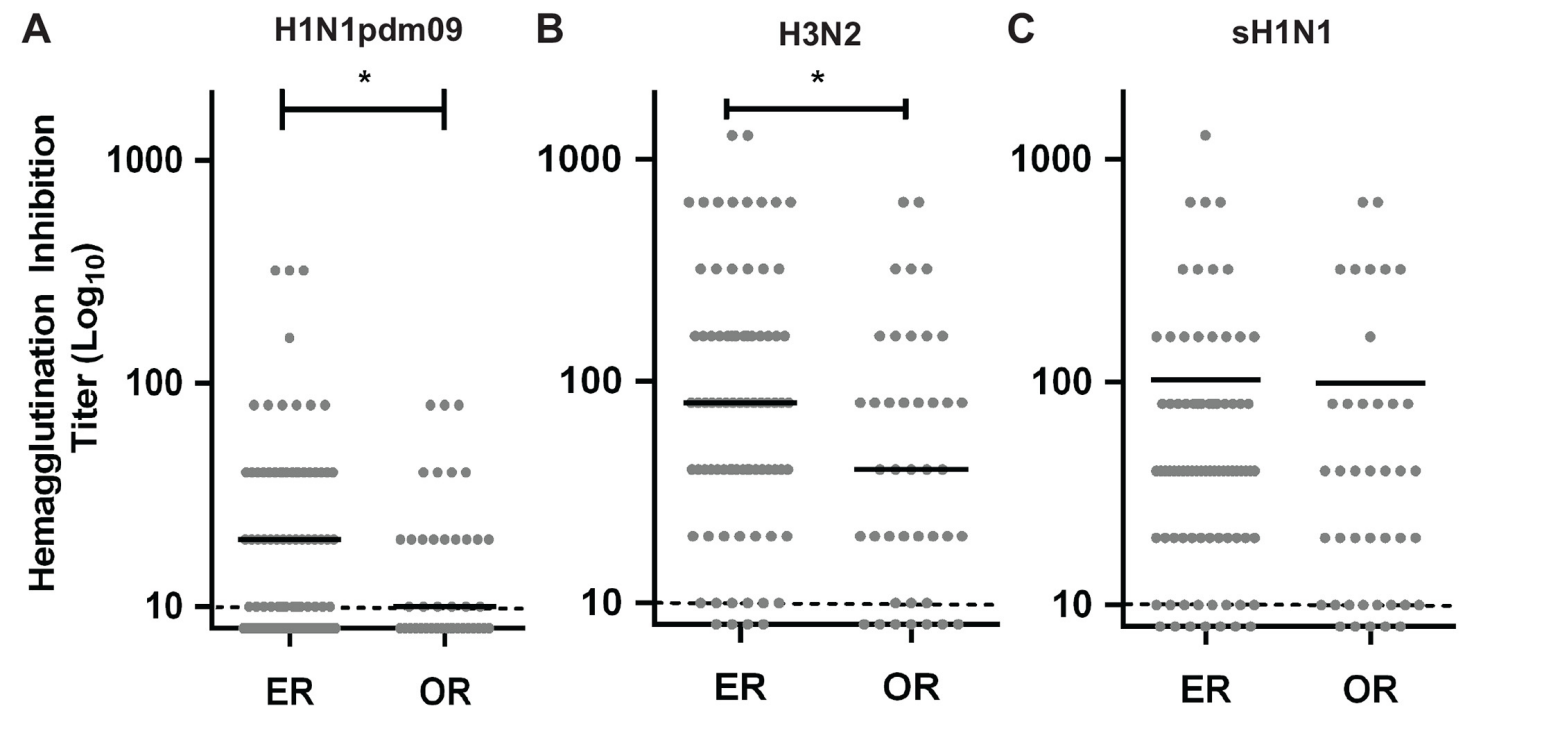
Seropositivity of health personnel to H1N1pdm2009 exposure during the pandemic outbreak in Chile. Post pandemic antibody titer of high risk (ER) and low risk health personnel (OR) at the PUC Clinical Hospital in Santiago of Chile, against **(A)** A/California/07/2009 H1N1pdm, **(B)** A/Brisbane/10/2007 H3N2 strains and **(C)** A/Brisbane/59/2007 seasonal H1N1. Serum samples were obtained at least 3 weeks after the pandemic period in Chile (after epidemiological week 33) and before H1N1pdm2009 vaccination (November to March of 2010). Grey dots depict individual HI titer, and black lines represent the mean titer for each group. Discontinuos grey line, indicates the assay threshold of detection set to 10 HIU. Statistical significance was determined by an Unpaired t-test with Welch's correction (* *p* 0.0405).

Interestingly, when we evaluated the antibody titer of the samples we found a statistically significant higher titer against the H1N1pdm2009 strain in the high-risk (ER) group than in the low risk personnel (OR, Fig 1A). While there was also a higher level of antibodies against the H3N2 seasonal strain, as expected there was no correlation with the titers seen against the H1N1pdm2009 (Fig 1A and 1B), since these two strains belong to vastly divergent antigenic groups (e.g. H1N1 belong to the Group 1 and H3N2 to Group 2). Similarly, there was no correlation between the titers seen for the seasonal H1N1 virus (Fig 1C). This confirmed that the seropositivity seen was specific for the H1N1pdm2009 strain and had no relation to background antibody titers against seasonal strains circulating in the population at the time or before the pandemic outbreak. Hence, this indicated that working in the ER resulted in the development of almost twice as much anti-influenza antibody titers than working in the OR (ER mean titer 36.8 ± 6.896, 95% CI 23.05 to 50.55; OR mean HI titer = 19.05 ± 3.033, 95% CI 12.92 to 25.17). Analysis also showed that the variances of both groups (ER and OR) were statically different (*p value* = 0.0001). Overall, these results indicate that working in a high-risk area of the hospital does not only increases the incidence of becoming infected during an influenza pandemic outbreak, but also suggests that these personnel might also be exposed to the virus more than once, resulting in higher levels of seropositivity rates and increased antibody titers.

## Discussion

During the 2009 influenza A virus pandemic outbreak many hospitals worldwide had an increased demand at their ER, resulting in a higher risk of exposure for health care personnel at those hospital units. In our hospital setting, 1 out of 3 members of the ER and 1 out of 7 members of the OR had evidence of infection by the H1N1pdm2009 strain after the end of its first wave in Chile. Considering an estimated 13% of population attack rate in Chile [17], both groups presented a higher exposure and seropositivity rate than the general population. This difference was even higher in the ER staff, whose probability of being seropositive was about 2.5 higher than staff working in the OR; and 3 times greater than the general Chilean population (37.8% versus 14.6%, respectively). These results suggest that, despite the use of infection control measures and the use of protective equipment, HCW in both hospital units have an increased risk of exposure to the virus during a pandemic outbreak and that this risk is even higher in the ER. This might be explained due to the increased demand of daily ER visits, which highly compromised the ability to separate patients in the waiting area. This could also result from possible breaks in the compliance of infection control measures by HCW. Similarly, OR personnel were probably more likely to interact or became in contact with an infected patient in the hospital (nosocomial infections) than people in the general population. Thus, once risk factors of health personnel are taken into account, our study resembles the findings seen in a hospital setting in Spain [11], during the first wave, but contrast the findings of two other studies in Australia and the United States, respectively, that did not find a substantial increased risk for infection to frontline health care workers [15, 16]. Moreover, our data are also similar to those found in previous studies that compared the prevalence of pandemic virus exposure between hospital personnel and control subjects or the general population, demonstrating an increased infection rate in hospital staff [5–14]. These contrasting findings, might be explained due to the different timing of the sampling, in the middle of the influenza season during the first wave in Chile, where one might expect a higher chance of transmission, versus the spring seasons of the US and Australia [15, 16] which might reduce contact and aerosol transmission. However, our findings also emphasize the value of evaluating risk factors in diverse clinical settings and countries in both hemispheres that reflect the reality of different health systems.

In this study we recruited 117 subjects on a voluntary basis, thus we cannot rule out to have inadvertently produced a bias in the final number of individuals recruited per subgroup. Nonetheless, we were able to find significant differences between the ER and OR personnel, which allowed elucidating factors that contribute to their risk of becoming infected in a pandemic setting. When we compared the chance of being seropositive between members of the ER and OR, the risk of infection differs according to the scenario and type of work. The increased prevalence observed in the ER staff might be explained by the greater contact at the emergency ward with ILI patients, many of them infected with the pandemic virus at that time. This observation was tested by adjusting for the association between possible confounding variables (pre-pandemic vaccination, use of clinical mask and adherence to hand hygiene during patient care), always obtaining a significantly higher risk in the absence of these variables (Table 2). These findings confirm the greater probability of acquiring the disease when working at a high-risk hospital unit [3, 5].

In addition, the antibody titers observed were specific against the H1N1pdm2009 strain, which is consistent with previous reports that indicated that antibodies against pre-pandemic seasonal strains do not cross-react with H1N1pdm2009 viruses[18, 20, 21]. The higher antibody titers observed in the ER personnel might be due to these individuals having secondary exposures to the virus (or potentially multiple exposures) during the pandemic outbreak. Thus, this increased antibody titers might be explained due to boosting. However, we cannot rule out that these individuals underwent a primary infection that resulted in higher titers. Nevertheless, many of these individuals did not present ILI and could have undergone one or more subclinical infections during the first wave (in the Southern Hemisphere). In fact, our study revealed that one third of the health personnel that was seropositive did not present ILI symptoms, and the majority of them (83%) were involved in direct patient care. Previous studies have shown that asymptomatic infection can be common and responsible of up to two thirds of influenza cases [4]. Hence, our data emphasize that asymptomatic infections must be considered when implementing additional and more effective measures to prevent the propagation of influenza virus infection [4, 17].

During an epidemic, HCW play an important role in providing health care for sick individuals. Nonetheless, health personnel can also become a significant source of transmission to patients needing health assistance, and even to other coworkers. Our results emphasize the importance of strengthening the preventive measures among HCW, considering their important role during an epidemic and/or pandemic period. Moreover, in our study half of the personnel tested that did not perform direct clinical duties also became seropositive by the end of the pandemic season. Therefore preventive practices must become extensive to all personnel that have any sort of interaction with patients even prior to the triage step. Unfortunately, these groups of people are often not considered during preventive campaigns in public health hospitals, including obligatory vaccination and the use of personal protective equipment, a scenario that should be re-considered due to their potential role in viral transmission to patients, their relatives or other health personnel. In fact, this study verified the utility of measures such as use of clinical mask and hand hygiene, which were associated with a decreased risk of becoming seropositive, in agreement to other investigations [3, 22–24], and therefore reinforce that these measures should be extensive to all personnel.

Overall, these results emphasize the need to promote the compliance of preventive and protective measures, in order to reduce the possibility of transmission among hospital personnel (including clerks and security guards) and patients. This is particularly relevant when health personnel are required to assist the high demand generated during a large epidemic or pandemic outbreak, and also to prevent them from becoming a source of infection to the patients at the clinic.
